# Lifestyle adaptations in cardiometabolic patients after COVID-19

**DOI:** 10.15649/cuidarte.3841

**Published:** 2024-08-23

**Authors:** Thamires Sales Macêdo, Nelson Miguel Galindo, Luana Eugenia de Andrade Siqueira Parente, Simone de Sousa Paiva, Joselany Áfio Caetano, Lívia Moreira Barros

**Affiliations:** 1 Universidade Federal do Ceará - UFC, Fortaleza, Ceará, Brazil. E-mail: thamiressales1998@outlook.com Universidade Federal do Ceará Universidade Federal do Ceará Fortaleza Ceará Brazil thamiressales1998@outlook.com; 2 Instituo Federal de Educação, Ciência e Tecnologia de Pernambuco - IFPE, Pesqueira, Pernambuco, Brazil. E-mail: nelsongalindont@hotmail.com Instituto Federal de Educação, Ciência e Tecnologia de Pernambuco Instituo Federal de Educação, Ciência e Tecnologia de Pernambuco Pesqueira Pernambuco Brazil nelsongalindont@hotmail.com; 3 Universidade da Integração Internacional da Lusofonia Afro-Brasileira - UNILAB, Redenção, Ceará, Brazil. E-mail: luanaeugenia12@hotmail.com Universidade da Integração Internacional da Lusofonia Afro-Brasileira Universidade da Integração Internacional da Lusofonia Afro-Brasileira Redenção Ceará Brazil luanaeugenia12@hotmail.com; 4 Universidad de Santiago de Chile - USACH, Chile. E-mail: simonesh.paiva@gmail.com Universidad de Santiago de Chile Universidad de Santiago de Chile Chile simonesh.paiva@gmail.com; 5 Universidade Federal do Ceará - UFC, Fortaleza, Ceará, Brazil. E-mail: joselany@ufc.br Universidade Federal do Ceará Universidade Federal do Ceará Fortaleza Ceará Brazil joselany@ufc.br; 6 Universidade da Integração Internacional da Lusofonia Afro-Brasileira - UNILAB, Redenção, Ceará, Brazil. E-mail: livia.moreirab@hotmail.com Universidade da Integração Internacional da Lusofonia Afro-Brasileira Universidade da Integração Internacional da Lusofonia Afro-Brasileira Redenção Ceará Brazil livia.moreirab@hotmail.com

**Keywords:** COVID-19, Chronic Disease, Health Promotion, Lifestyle, COVID-19, Enfermedad Crónica, Promoción de la Salud, Estilo de Vida, COVID-19, Doença Crônica, Promoção da Saúde, Estilo de Vida

## Abstract

**Introduction:**

Numerous factors contribute over time to changes in lifestyle behaviors, and the COVID-19 pandemic not only altered individuals' routines but also influenced the factors affecting their chronic conditions.

**Objective:**

To understand the perception of patients with cardiometabolic diseases regarding adaptations to their lifestyle after contracting COVID-19.

**Materials and methods:**

This exploratory study with a qualitative approach was conducted with nine patients diagnosed with cardiometabolic diseases who tested positive for COVID-19. Data collection involved semi-structured audio-recorded interviews, which were transcribed and processed using IRAMUTEQ software.

**Results:**

The collective discourse revealed that mass contamination by the coronavirus and subsequent Long COVID triggered significant fears and anxieties, as well as increases in muscle pain, fatigue, and difficulties in maintaining blood pressure and/or glycemic control. These issues directly impacted the daily routines of infected patients. However, there was also an awakening due to changes in lifestyle.

**Discussion:**

The complexity of the experiences reported by infected patients influenced their desire to adopt a healthy lifestyle and seek knowledge.

**Conclusion:**

The number of patients affected by COVID-19 in its acute and long phases is concerning for both society and health professionals, highlighting the need to expand strategies aimed at quality care and ensuring adequate monitoring across different spheres of care.

## Resumen

**Introducción:**

Innumerables factores contribuyen en el tiempo a cambiar conductas relacionadas con el estilo de vida, la pandemia de COVID-19 no solo alteró las rutinas de los individuos, sino que también hizo cambios en los factores que influyen en sus condiciones crónicas.

**Objetivo:**

Conocer la percepción de los pacientes con enfermedades cardiometabólicas, con relación a las adaptaciones en su estilo de vida después del contagio por COVID-19.

**Materiales y Métodos:**

Estudio exploratorio con enfoque cualitativo, realizado con nueve pacientes diagnosticados con enfermedades cardiometabólicas y que resultaron positivos a COVID-19. La recolección se realizó a partir de entrevistas grabadas en audio semiestructurado y su contenido fue transcrito y procesado en el software IRAMUTEQ.

**Resultados:**

El discurso colectivo mostró que la contaminación masiva por coronavirus y, posteriormente, Long-COVID desencadenó muchos miedos y angustias, además de un aumento de los dolores musculares, cansancio y la falta de control de la presión arterial y/o la glucemia, impactando directamente en la rutina de vida diaria de los pacientes infectados, pero también hubo un despertar por cambios en el estilo de vida.

**Discusión:**

La complejidad de las experiencias relatadas por los pacientes infectados incide en el deseo de adoptar un estilo de vida saludable y la búsqueda de conocimiento.

**Conclusiones:**

El número de pacientes afectados por COVID-19 en sus fases aguda y prolongada es preocupante para la sociedad y los profesionales de la salud, siendo necesario ampliar estrategias que apunten a la calidad de la atención, buscando un adecuado seguimiento en las diferentes esferas de la atención.

## Resumo

**Introdução:**

Inúmeros fatores contribuem ao longo do tempo para a mudança de comportamentos relacionados ao estilo de vida, a pandemia de COVID-19 não apenas alterou as rotinas dos indivíduos, mas também provocou alterações nos fatores que influenciam suas condições crônicas.

**Objetivo:**

Conhecer a percepção de pacientes com doenças cardiometabólicas, em relação às adaptações no seu estilo de vida após a infecção pela COVID-19.

**Materiais e Métodos:**

Estudo exploratório com abordagem qualitativa, realizado com nove pacientes com diagnóstico de doenças cardiometabólicas e que testaram positivo para COVID-19. A coleta foi realizada a partir de entrevistas gravadas em áudio semiestruturado e seu conteúdo foi transcrito e processado no software IRAMUTEQ.

**Resultados:**

O discurso coletivo mostrou que a contaminação massiva pelo coronavírus e, posteriormente, pelo Long-COVID desencadeou muitos medos e ansiedades, além do aumento de dores musculares, fadiga e descontrole da pressão arterial e/ou glicemia, impactando diretamente no cotidiano. rotina de vida dos pacientes infectados, mas também houve um despertar devido às mudanças no estilo de vida.

**Discussão:**

A complexidade das experiências relatadas pelos pacientes infectados afeta o desejo de adoção de um estilo de vida saudável e a busca por conhecimento.

**Conclusões:**

O número de pacientes acometidos pela COVID-19 em suas fases aguda e prolongada é preocupante para a sociedade e para os profissionais de saúde, tornando necessária a ampliação de estratégias que visem a qualidade da assistência, buscando um acompanhamento adequado nas diferentes esferas de atenção.

## Introduction

Lifestyle (LS) is a factor that impacts individuals’ quality of life (QoL) and can be defined as a set of modifiable behaviors that influence a person’s attitudes, values, and opportunities, becoming an important determinant of the health of individuals, groups, and communities^1^.

Over time, numerous factors contribute to completely changing behaviors related to LS^2^. It is known that diet, physical activity, sleep behaviors, cigarette consumption, alcohol intake, and stress are independent risk factors that can lead to Cardiometabolic Diseases (CMD), including type 2 diabetes mellitus, obesity, and cardiovascular disorders^2^^, ^^3^.

The public health crisis generated by the COVID-19 pandemic not only altered individuals’ routines but also brought changes to their LS. Patients infected with the novel coronavirus were susceptible to various complications related to the cardiovascular system, gastrointestinal tract, nervous system, and other systems^4^.

However, after the acute phase of the coronavirus, persistent symptoms such as fatigue, muscle pain, malaise, myalgia, depression, anxiety, and headaches were observed for weeks or months. These symptoms affected various body systems and caused psychological effects that contributed to reduced productivity, difficulty integrating into society, and challenges in returning to daily activities and work, known as the effects of Long COVID^5^.

A quantitative cohort study with 313 adults conducted in Mexico explored the dimensions related to a healthy lifestyle and its relationship with demographic variables during COVID-19 confinement^6^. The study demonstrated low levels of health responsibility linked to physical activity and stress management. Furthermore, patients with persistent COVID reported frequent symptoms such as fatigue, memory loss, shortness of breath, decreased mood levels, well-being, anxiety, depression, post-traumatic stress disorder, and insomnia^7^.

Despite this, much remains to be investigated regarding the risk factors and the development of specific symptoms associated with post-COVID-19^8^. There is a need to understand the perceptions and health issues generated among patients with CMD and develop strategies aimed at restoring a healthy LS. Therefore, this study aims to understand the perceptions of patients with CMD regarding adaptations to their lifestyle after contracting COVID-19.

## Materials and Methods

An exploratory study with a qualitative approach was conducted from March to June 2023. The study was carried out in the Basic Health Units (UBS) of the municipality of Crateús, located in the interior of the state of Ceará, Brazil, recognized as a Regional Center for Primary Care.

Crateús is located 353 km from the state capital and has a total of 26 UBS, 12 of which are in the city center. Services are provided from Monday to Friday, from 7:00 AM to 5:00 PM, according to population demands and in conjunction with the primary care programs of the Unified Health System (SUS).

The population comprised individuals with a clinical diagnosis of CMD, such as hypertension, type 2 diabetes mellitus, obesity, or cardiovascular disease, associated with a positive laboratory diagnosis of COVID-19. The sampling was non-probabilistic and intentional, as these individuals may have faced challenging situations after being affected by the coronavirus due to its high potential for clinical deterioration when already presenting comorbidities.

The inclusion criteria were being 18 years or older, having a diagnosis of CMD, having had a positive laboratory test for COVID-19, and being under follow-up at the health unit. The exclusion criterion was exhibiting any clinical instability during the interview. Consequently, nine participants were included in the study sample, as the objective was achieved and data saturation was reached^9^.

Recruitment was conducted in the UBS waiting room. Subjects were approached individually and invited to participate in the research, followed by the signing of the Informed Consent (IC). All approached patients who met the inclusion criteria agreed to participate.

Data were collected through semi-structured interviews conducted by researchers of both genders, with academic and professional backgrounds in health and clinical experience. A reserved area within the unit, such as an office or the most isolated hallway, was used for the interviews, which lasted an average of 20 minutes.

The guiding instrument was divided into two parts: the first included clinical and epidemiological characterization with variables such as gender, age, origin, monthly income, comorbidity, and possible hospitalization. The second part presented the trigger question: “After the COVID-19 diagnosis, did you experience changes in your daily routine?” The responses were recorded using a recording application on the researchers’ cell phones.

The audio-recorded data were fully transcribed and processed using the *Interface de R pour les Analyses Multidimensionnelles de Textes et de Questionnaires* (IRAMUTEQ) software version 0.7 Alpha 2.3.3.1. The software, anchored to the R program, divides the textual content into segments and allows for the measurement of word frequency and multivariate analysis through Descending Hierarchical Classification (DHC). In this multivariate analysis, segments are grouped into classes using the Chi-squared test so that the classes consist of segments that are similar and different from those composing other classes.

The visual representation of the relationship between the classes is presented by the software in the form of a dendrogram. Data processing by IRAMUTEQ was compatible, as the transcription consisted of 73 segments, of which 76.71% were used for analysis. All collected data are available for free access and consultation on Figshare^10^.

Confidentiality and anonymity of the participants were respected, and the identification of those involved in the study was presented using the letter “P”, followed by a numerical sequence according to the order of the interviews.

The research adhered to the guidelines and regulatory standards for research involving human subjects, as outlined in Resolution 466/12 of the National Health Council (CNS) of the Ministry of Health. It was submitted to and approved by the Research Ethics Committee of the University of International Integration of the Afro-Brazilian Lusophony under Opinion no. 4.429.720.

## Results

Among the nine patients who participated in the study, females were predominant 66.66% (n=06). The age group ranged between 45 and 65 years. Regarding the diagnosis of any of the CMDs, 77.77% (n=07) had hypertension, 55.55% (n=5) had diabetes mellitus, 44.44% (n=4) had both conditions, and 33.33% (n=3) were obese. All had a positive laboratory test for COVID-19 during the pandemic, and 44.44% (n=4) required hospitalization.

The collective discourse revealed that mass infection by the coronavirus and subsequently experiencing Long COVID triggered negative consequences for CMDs. Additionally, many fears and anxieties related to the new infection were identified. However, the narratives also showed a desire to change daily habits, such as adopting a healthy diet, engaging in physical exercise, seeking clinical follow-up from a multidisciplinary team, and continuing with distancing and barrier practices to avoid contamination.

Patients' perceptions are central in their accounts and are intertwined with the difficulties faced during and after COVID-19. These perceptions also explore the need to transform awareness about the benefits of healthy practices and habits.

**Figure 1:**
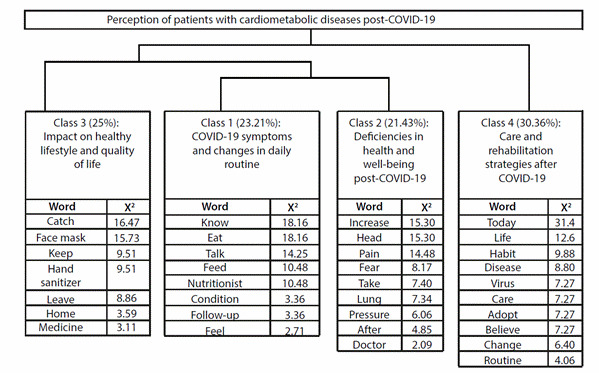
IRAMUTEQ dendrogram. Crateús, Ceará, Brazil. 2024

Through Descending Hierarchical Classification (DHC), a dendrogram was generated in IRAMUTEQ, dividing the 73 segments into four classes, as shown in (Figure 1).

Through the content analysis of the data, four categories emerged: symptoms of COVID-19 and changes in daily routine; Health and well-being deficiencies post-COVID-19; Impacts on healthy lifestyle and quality of life; and Care and rehabilitation strategies after COVID-19.

### COVID-19 symptoms and changes in daily routine

In response to a COVID-19 diagnosis, experiences with classic symptoms such as anosmia, fatigue, sneezing, headache, fever, and loss of appetite are reported. The narratives indicate that these symptoms were exacerbated in the initial days of the illness, directly impacting on daily routines.

Additionally, feelings of fear regarding the presented symptoms are identified, particularly related to the concern of transmitting the new virus to family members.

*"I went 15 days without tasting or smelling my food. I drank more orange juice because that way I knew I was getting nourishment since I couldn't eat the food itself." (P3)*

*"I only had trouble cooking because I couldn't smell things properly." (P2)*

*"I was afraid of getting infected again or contaminating my family. My wife gave me my meals, and I've also had weakness in my legs for about three months now." (P7)*

In response to the symptoms of the illness, a constant concern among participants was the pursuit of additional knowledge about COVID-19, particularly regarding the necessary guidance following the remission of the disease.

*"It never hurts to know, so if my blood pressure changes more, I wanted to know the changes and the aftermath." (P1)*

*"As a patient, I must suggest that health establishments adopt follow-up measures in the community for these people. For example, we had many people who had COVID and were there at the health center seeking care. After one comes home, there was no professional who came to give a talk, to advise the patient to go back to the clinic to check their blood pressure, how it is after the illness." (P9)*

In addition to the physical symptoms, the presence of Long COVID also elicited feelings and emotions such as fear, insecurity, mood swings, uncertainty about future events, and a desire for change.

*"So, all of this left this aftermath, left this fear. So, I had to change." (P9)*

*"My blood pressure was altered, at that moment I didn't leave the room, inside the house all the time because I was afraid of getting infected again." (P7)*

*"I'm scared because the doctor warned me: you've already had COVID twice and you're hypertensive; if I already had 90% care, I have to be more than 100% because I'm diabetic and have high blood pressure, it's very dangerous, and since it reached my lung and I was at 25% and very bad, I get scared." (P4)*

### Post-COVID-19 health and well-being deficiencies

Reports in this category reveal the negative experiences associated with chronicity and Long COVID. The narratives reference issues ranging from increased muscle pain, headaches, and fatigue to symptoms related to blood pressure and/or glycemic control.

*"The headaches, leg pain, fatigue, even the blood pressure and diabetes got worse." (P4)*

*"Yes, I had trouble hearing, here and there I feel dizzy, headache because my blood pressure is going up, but I control it by taking my medication." (P1)*

*"I feel very tired, I had to sit down and hold off on doing things and then start again, the fatigue was too much, and sometimes it's still noticeable." (P5)*

*"I was only more compromised by the lack of smell; I haven't smelled anything since COVID times, it makes cooking complicated, sometimes the food burns right under my nose, and I don't smell anything." (P2)*

*"I'm sorry, we feel tired, our bodies aren't the same, we get weaker, the disease has weakened people." (P3)*

Additionally, some accounts describe difficulties in managing everyday situations after the infection, with individuals seeking assistance from their support network to cope with physical limitations and emotional care.

*"I work, thank God, it's just that sometimes I get tired, and that's why sometimes my husband takes me to work on his bicycle." (P1)*

*"Yes, I have help with cleaning the house and ironing clothes, my difficulty has increased a lot, and it causes me faster fatigue and back pain." (P6)*

*"It changed because I lost some friends, and my children also got infected because it was my kids who took care of me." (P3)*

*"I don't know if it's because of COVID, my profession was a bicycle mechanic, then after I recovered, it was already the end of the year, I tried to go back, and I couldn't go back to work." (P7)*

### Impact on healthy lifestyle and quality of life

The clinical deterioration highlighted the need to adopt new attitudes and behaviors regarding health, such as implementing preventive measures and controlling the coronavirus, including social distancing and the use of masks to prevent virus transmission. Participants also expressed a desire to adopt healthy lifestyle habits, such as engaging in physical activity and adhering to pharmacological treatment for CMD.

*"I'm very careful, I don't take off my mask for anything, I wash my hands well, I put alcohol on my hands, and whether I go somewhere or come here, I don't take off my mask for anything or touch anything." (P2)*

*"I had to change my habits, like hygiene, with attention, and today I completely changed my way of living, with more care, with more attention." (P5)*

*"I wear a mask in most places, try to respect social distancing, use hand sanitizer, try to take my medication." (P6)*

*"I take several medications throughout the day to prevent heart diseases, and this has also changed my routine." (P9)*

*"I started walking as a way to get out of a sedentary lifestyle. Every day, early in the morning, I would go for this walk." (P9)*

*"I already exercise and eat well." (P2)*

However, some narratives also reveal a reluctance to change lifestyle habits despite the negative impacts that the viral infection prompted them to develop.

*"The physical activity I used to do was walking, but out of laziness, I don't go anymore." (P7)*

*"I'm not being careful, staying the same way I arrived. The food is the same. The routine of sometimes having a cachaça is the same." (P8)*

### Care and rehabilitation strategies after COVID-19

Based on their experience of a COVID-19 diagnosis and the need for social isolation, participants emphasized the importance of health care in pursuing well-being and quality of life. They highlighted the necessity of increasing knowledge about their CMD, as well as incorporating healthy lifestyle practices, ongoing follow-up with a multidisciplinary team, proper food choices, environmental hygiene, physical activity, and stress management.

*"I think today I live more attentively, not just to this virus but also to the other preexisting diseases." (P9)*

*"It's about knowing more about diabetes and these diseases we have, nutrition, the physical exercise we do, because these conversations are good and good for our mind, because we get older and forget things." (P4)*

*"My routine today is here, I would say with a lot of attention, the change in habits was constant in my life, I had to change the life I had to protect myself more." (P9)*

*"I also do follow-ups with the nutritionist." (P5)*

*"We know it's important to have a good diet, so knowing more about nutrition, as the nutritionist says, or with the psychologist, and knowing more things because it's important to continue the follow-up." (P1)*

*"Now I want to be referred to physiotherapy to see if I can improve my pain." (P6)*

## Discussion

This study captured the complexity of the experiences reported by patients with CMDs infected with COVID-19 who continue to present specific symptoms classified as Long COVID or persistent COVID. Furthermore, it indicates their willingness to make lifestyle changes towards adopting healthier habits and acquiring specific knowledge about their chronic condition to improve their quality of life and avoid complications associated with potential comorbidities.

Among the analyzed narratives, all participants highlighted that symptoms during the acute phase of COVID-19 and Long COVID hinder and complicate the performance of daily activities and the return to work, which aligns with a qualitative study conducted in India, showing negative impacts on financial and work performance^11^. Similarly, the new virus negatively affected the daily activities of 1.6 million patients in the United Kingdom, where they rated their performance as highly limited^12^.

The results of a qualitative study established that some individuals with Long COVID have difficulty returning to pre-infection routines and activities due to the persistence of symptoms related to physical and mental health, which impede daily functioning^13^.

Moreover, living with a CMD has heightened fear and anxiety, as published reports have confirmed that patients with COVID-19 and pre-existing chronic conditions experience worse health outcomes and higher mortality rates compared to those without such conditions^14^. Longitudinal studies have demonstrated that these patients may experience exacerbations of certain neuropsychiatric symptoms such as anxiety, depression, insomnia, and cognitive impairments^15^.

Insecurity and fear are conceptualized in studies describing infection-related episodic disability, where physical and cognitive health issues following COVID-19 fluctuate during symptom outbreaks-i.e., bad days followed by periods of controlled, fewer symptoms^16^.

Therefore, it can be noted that the most devastating effects occur during Long COVID. Currently, there are no established pathophysiological mechanisms or appropriate therapeutic options for this period^17^. A study conducted with 2.1 million people in the United Kingdom on the prevalence of persistent symptoms after coronavirus infection emphasizes that fatigue was the most reported symptom (71%), followed by difficulty concentrating (49%), shortness of breath (47%), and muscle pain (46%)^18^.

This directly and adversely impacts a healthy lifestyle, representing an unprecedented challenge for public health^19^. As observed in the present study, despite the desire to implement healthy lifestyle habits into daily routines, there were still reports of interruptions in pharmacological treatment, decreased physical activity, poor diet, and alterations in blood pressure and glycemic levels, which may drastically affect the quality of life for this population.

Consequently, the demand for healthcare services decreased due to fear of contracting new diseases and worsening their clinical condition, impacting interpersonal relationships and stress management^20^. However, there has been a growing emphasis on seeking simple strategies to cope with Long COVID, such as establishing new healthy habits, social and mental support, setting healthy goals, and returning to healthcare services.

Changes in mindset to pursue healthy habits were described by 47 patients in a qualitative study conducted in Canada as essential for coping with their situation, recognizing the need for optimism, and acceptance, seeking qualified professionals, and gradually returning to activities requiring more effort^21^.

For this reason, it is crucial to implement a health promotion agenda and strengthen Primary Health Care (PHC) to address the impacts generated by the pandemic, especially concerning the maintenance of comprehensive follow-up actions for patients with CMDs^22^.

Integrating the quality of individual and collective care is also necessary to uphold the principles of accessibility, continuity, coordination of care, community, and family-focused approaches. Nurses play a significant role in community outreach and health monitoring, performing a crucial role in the care network, as well as in managing and continuing post-infection care^23^.

In this context, healthcare professionals should engage in planning effective strategies to respond to Long COVID, ensuring the right to health for patients with CMDs affected by COVID-19, their families, and caregivers.

Limitations of this study include the fact that data were collected only from SUS users in the interior of northeastern Brazil, which may not reflect the situation in other regions, and that no nursing or psychological theories were used to explain the studied phenomenon.

## Conclusion

In conclusion, it is important to note that participants reported experiencing various symptoms over the months following a positive COVID-19 diagnosis, which impacted their daily routines. Despite this, they demonstrate a strong desire to seek new knowledge about their chronic conditions and to improve their lifestyle habits. Furthermore, the number of patients affected by COVID-19 in both its acute and prolonged phases is concerning for society, particularly when the infection occurs in patients with CMDs.

Therefore, to ensure appropriate follow-up in this new reality, it is essential to enhance and expand healthcare strategies focused on quality care, where nurses play a crucial role as mediators to encourage the necessary changes and adaptations to improve the quality and lifestyle of these patients.

Additionally, it is recommended to conduct further studies aimed at developing evidence-based technologies to enhance the care of individuals with CMDs, in order to support healthcare professionals and mitigate the impacts generated throughout the pandemic, as well as to ensure improvements in quality of life.
